# Allocation of Nitrogen and Carbon Is Regulated by Nodulation and Mycorrhizal Networks in Soybean/Maize Intercropping System

**DOI:** 10.3389/fpls.2016.01901

**Published:** 2016-12-16

**Authors:** Guihua Wang, Lichao Sheng, Dan Zhao, Jiandong Sheng, Xiurong Wang, Hong Liao

**Affiliations:** ^1^State Key Laboratory for Conservation and Utilization of Subtropical Agro-bioresources, Root Biology Center, South China Agricultural UniversityGuangzhou, China; ^2^Xinjiang Key Laboratory of Soil and Plant Ecological Processes, College of Grassland and Environmental Sciences, Xinjiang Agricultural UniversityUrumqi, China; ^3^Root Biology Center, Fujian Agriculture and Forestry UniversityFuzhou, China

**Keywords:** arbuscular mycorrhizal inoculation, carbon allocation, common mycorrhizal network, ^15^N labeling, nitrogen transfer, rhizobium inoculation, soybean/maize intercropping, ^13^C labeling

## Abstract

Soybean/maize intercropping has remarkable advantages in increasing crop yield and nitrogen (N) efficiency. However, little is known about the contributions of rhizobia or arbuscular mycorrhizal fungi (AMF) to yield increases and N acquisition in the intercropping system. Plus, the mechanisms controlling carbon (C) and N allocation in intercropping systems remain unsettled. In the present study, a greenhouse experiment combined with ^15^N and ^13^C labeling was conducted using various inoculation and nutrient treatments. The results showed that co-inoculation with AMF and rhizobia dramatically increased biomass and N content of soybean and maize, and moderate application of N and phosphorus largely amplified the effect of co-inoculation. Maize had a competitive advantage over soybean only under co-inoculation and moderate nutrient availability conditions, indicating that the effects of AMF and rhizobia in intercropping systems are closely related to nutrient status. Results from ^15^N labeling showed that the amount of N transferred from soybean to maize in co-inoculations was 54% higher than that with AMF inoculation alone, with this increased N transfer partly resulting from symbiotic N fixation. The results from ^13^C labeling showed that ^13^C content increased in maize shoots and decreased in soybean roots with AMF inoculation compared to uninoculated controls. Yet, with co-inoculation, ^13^C content increased in soybean. These results indicate that photosynthate assimilation is stimulated by AM symbiosis in maize and rhizobial symbiosis in soybean, but AMF inoculation leads to soybean investing more carbon than maize into common mycorrhizal networks (CMNs). Overall, the results herein demonstrate that the growth advantage of maize when intercropped with soybean is due to acquisition of N by maize via CMNs while this crop contributes less C into CMNs than soybean under co-inoculation conditions.

## Introduction

Intercropping is an essential element of agricultural sustainability, and widely practiced in tropical, subtropical, and temperate areas (Maffei and Mucciarelli, 2003; Brooker et al., 2015). One common intercropping scheme is to pair a cereal crop with a legume that can supply N through biological N_2_ fixation. Yield advantages of intercropping legumes with non-N_2_ fixing crops have been found in many intercropping systems, including ryegrass-subclover (Ledgard et al., 1985), rice-peanut (Shen and Chu, 2004), wheat-faba bean (Xiao et al., 2004), Millet-cowpea (Laberge et al., 2011), rapeseed-faba bean (Jamont et al., 2013), and maize-soybean (Tang et al., 2005; Meng et al., 2015). Despite the prevalence of such cropping systems, the mechanisms underlying yield advantages of intercropping compared with monocropping systems have not been fully explored.

It has been reported that intercropping advantages can be explained by above-ground plant–plant interaction effects on light absorption, maintenance of optimal temperatures and space, or below-ground interactions, including root–root interactions, and interactions between roots and beneficial soil microbes (Brooker et al., 2015; Li et al., 2016; Xue et al., 2016). Root–root interactions may benefit intercropped plants in several ways. In the soybean/maize intercropping system, maize yields are greater when intercropped with soybean genotypes with shallow roots than with deep root architectures (Tang et al., 2005). Intercropped plants may also benefit from root exudates of neighboring plants. In the fababean/maize intercropping system, the release of root exudates by fababean promotes acidification of the rhizosphere and enhances P mobilization and acquisition in maize (Li et al., 2007), while root exudates from maize stimulate flavonoid synthesis and nitrogen fixation in fababean (Li et al., 2016).

Interactions between roots and beneficial soil microbes also play very important roles in increasing intercropping advantages. Beneficial soil microbes, such as AMF, can promote the mineralization and mobilization of nutrients, and more importantly provide pathways for transfer of nutrients through CMNs between intercropped crop species (Newman et al., 1994; Xiao et al., 2004; He et al., 2005; Walder et al., 2012). The CMNs can also facilitate transfer of carbon, water, defense signals and allelochemicals between cocultivated crops (Meding and Zasoski, 2008; Mikkelsen et al., 2008; Simard et al., 2012; Walder et al., 2012; Deslippe et al., 2016). Another beneficial soil microbe, *Rhizobium*, is able to interact with roots of legumes to form N-fixing nodules, which increases the supply of N in intercropping systems. It has been demonstrated that intercropped crops may benefit from symbiotic associations with both rhizobia and AMF. Nitrogen fixed by legume plants can be transferred to non-legumes via root contact or CMNs in many intercropping systems (Xiao et al., 2004; Sierra and Nygren, 2006; Laberge et al., 2011; Meng et al., 2015). However, net N transfer from legumes to non-legumes through CMNs has been sparsely documented.

Plants transfer as much as 4–16% of photosynthetically fixed carbon (C) to AM and rhizobial symbionts in order to maintain an extensive hyphal network and nodule growth (Wright et al., 1998; Kaschuk et al., 2009). There is evidence the benefits of CMN, as well as the C costs of AMF and rhizobia, vary among cocultivated plants, and depend on the AMF and plant species involved, soil nutrient supply and growth conditions (Kaschuk et al., 2009; Walder et al., 2012; Fellbaum et al., 2014). Meanwhile, carbon sink strengths of rhizobial and AM symbioses can also stimulate photosynthetic carbon assimilation (Kaschuk et al., 2009). However, until now, how the partitioning of photosynthate among intercropped legumes and non-legumes is regulated by beneficial soil microbes has been largely unexplored. In addition, mechanisms controlling carbon allocation among intercropped crops connected by a CMN are unsettled.

Soybean (*Glycine max* L.) and maize (*Zea mays* L.) are important food crops. Soybean/maize intercropping is one of the most common agricultural cultivation systems in China. Intercropping soybean can improve maize growth in the field (Tang et al., 2005). However, exactly how soybean promotes maize growth and the potential roles of AM and rhizobial symbiosis remain unclear. Therefore, we hypothesized that the growth advantage of maize when intercropped with soybean is partly due to allocation of carbon and nitrogen regulated by nodulation and mycorrhizal networks. In the present study, soybean and maize grown in an experimental intercropping system were inoculated with AMF and/or rhizobia in order to explore the contributions of AMF and rhizobia in this intercropping system, and to quantify nitrogen transfer and carbon allocation via ^15^N and ^13^C isotope labeling.

## Materials and Methods

### Experimental Materials

The two host plants used were the soybean variety HN89 and the maize variety Tiannuo. The AM fungal partner was *Rhizophagus irregularis* (DAOM 197198; accession no. AUPC00000000; Tisserant et al., 2013; Wang et al., 2016), and the rhizobial isolate was belonging to *Bradyrhizobium elkanii* strain BXYD3.

### Experimental Design

One greenhouse experiment and one growth chamber labeling experiment were conducted. The greenhouse experiment was carried out to check soybean growth performance in response to the varying N and P levels, and rhizobium and AM inoculation treatments, which included four N and P treatments (LN and P, LNLP, 265 μM N and 25 μM P; LN and medium P, LNMP, 265 μM N and 250 μM P; MN and LP, MNLP, 2650 μM N and 25 μM P; MN and P MNMP, 2650 μM N and 250 μM P), two culture modes (monoculture and intercropping) and three combinations of AM fungal and rhizobium inoculation treatments, including an uninoculated control (-A-R), AM fungal inoculation (+A-R), and inoculation of both the AMF and rhizobium (+A+R). There were four replicates of each treatment. The growth chamber labeling experiment had one N and P treatment (530 μM N and 50 μM P) with two cropping systems (monoculture and intercropping) and three combinations of AM fungal and rhizobium inoculation (-A-R, +A-R and +A+R) in order to explore N transfer and C allocation in CMNs. N and P concentrations were increased compared to LNLP levels in greenhouse experiment, but had not been increased to those at MNMP levels since the plants in MNMP were too large to handle for ^13^C labeling. There were six replicates of each treatment. Three were used for ^15^N and ^13^C labeling, and the other three were used as controls to examine natural ^15^N and ^13^C abundances, and adjust for the ^15^N and ^13^C abundance of plant samples from the background contribution.

### Growth Conditions

A two-compartment growth system was employed for both the greenhouse experiment and the growth chamber labeling experiment (**Supplementary Figure S1**). Soybean and maize plants were grown side by side in the two-compartment system constructed out of perspex boxes (13 cm × 12 cm × 12 cm). The two compartments were evenly separated by a perforated perspex divider (0.5 cm wide) to form a 0.5 cm air gap in order to prevent ion diffusion between compartments. Every side of the divider was enclosed by a 30 μm nylon mesh that allowed only fungal hyphal connection while preventing root contacts. Every compartment was filled with 1 kg of growth substrate consisting of a 4:1 mix of sand and soil that was autoclaved in two 1 h 121°C treatments in order to inhibit microbial contamination. The growth substrate had a pH of 5.0 when mixed with water, along with 1.2 mg kg^-1^ of available P as determined by the Bray II method, and 47.2 mg kg^-1^ of available N as determined by the alkali-hydrolyzed diffusion method.

In the greenhouse experiment, plants were grown under natural light in the intensity range of 500–1800 μmol m^-2^ s^-1^. Diurnal air temperatures ranged consistently between 20 and 28°C. Prior to planting, 10% dry inoculum was incorporated into the growth substrate. The inoculum consisted of colonized millet root fragments and a soil:sand mix containing the spores and extraradical hyphae of *R. irregularis*. The uninoculated control also incorporated the 10% soil:sand mix from millet grown without mycorrhizal inoculation. Soybean seedlings were inoculated with 2 mL rhizobium solution 1 week after germination (Wang et al., 2011). Plants were watered with soybean nutrient solution (Zhang et al., 2015) containing different N and P concentrations every week throughout the growth period.

In the growth chamber labeling experiment, plants were grown in a walk-in growth chamber with a 16 h photoperiod, a 24°C/22°C day/night temperature cycle, and a light intensity of 455 μmol m^-2^ s^-1^. After germination, seedlings were inoculated by adding 500 spores of *R. irregularis* into the soil close to the roots. After 1 week, soybean seedlings were inoculated with 2 mL of rhizobium solution (Wang et al., 2011). Plants were watered every week with soybean nutrient solution containing 50 μM P and 530 μM N.

### ^15^N and ^13^C Labeling

In the growth chamber labeling experiment, ^15^N labeling was conducted according to Xiao et al. (2004) with some modifications. Six weeks after planting, soybean plants were labeled through petiole injection with (^15^NH_4_)_2_SO_4_ enriched with 99% ^15^N. Ten microliter of 88 mM (^15^NH_4_)_2_SO_4_ solution was injected through a 25 μL syringe each day for 9 days in succession, and thereby supplying every soybean plant with a total of 0.22 mg ^15^N.

^13^C labeling was conducted according to Lu et al. (2002a) with some modifications. Briefly, at the time of labeling, the surface of the perspex boxes was sealed with plastic film with a narrow slot for the shoot to ensure the isolation of the shoots from the roots, and to create a shoot-labeling box. The Perspex boxes were transferred to transparent labeling chambers (50 cm × 52 cm × 50 cm), which was also sealed to prevent ^13^CO_2_ leakage. ^13^CO_2_ gas inside the chamber was generated by adding lactic acid into vial of Ba^13^CO_3_ (98% ^13^C) with a syringe. The mean ^13^CO_2_ concentrations were 400–450 μL L^-1^ in the chamber. Four fans were used to recirculate the air within the closed labeling chambers. Plants were labeled at midmorning for 6 h, and then taken out from the chamber for 3 days before harvesting.

### Harvest

After 50 days, plants in the greenhouse experiment were harvested. Shoots, roots, and nodules were separated for determining plant dry weight, plant N and P concentrations, nodule number, and nodule fresh weight. One fresh root subsample was weighed and then cleared with 10% KOH solution and stained with 5% ink-vinegar solution for assessing AM colonization (Wang et al., 2016). Nitrogen and P concentrations were measured using a San++ SKALAR continuous flow analyzer (Skalar, The Netherlands) after acid digestion.

In the growth chamber labeling experiment, plants were harvested 3 days after ^13^C labeling, and the shoots, roots and nodules were separated. The roots were washed, weighed, and divided into two portions; one subsample was used for determination of AM colonization (Wang et al., 2016); and the rest of the samples were dried to determine ^15^N and ^13^C concentrations, along with N, C, and P concentrations. The concentrations of ^15^N and ^13^C in the shoots, roots, and nodules were determined using Delta V Advantage isotope ratio mass spectrometry (Thermo Finnigan, German). Total carbon was determined using a Euro EA3000 single elemental analyzer (Euro Vector, Italy).

### ^15^N Transfer Calculations

N transfer was calculated as follows (He et al., 2009; Meng et al., 2015):

Percentage N transfer (% N_transfer_):

%Ntransfer=Ncontentmaize15×100/(Ncontentmaize15+Ncontentsoybean15)

Where ^15^N content = atom% ^15^N excess × total N; The atom% ^15^N excess was calculated by subtracting the natural abundance of ^15^N in unlabeled soybean or maize from the measured atom% ^15^N of labeled soybean or maize.

Amount of N transferred from soybean to maize (N_transferred_, mg plant^-1^):

$${\text{N}}_{\text{transferred}}\,=\%{\text{N}}_{\text{transfer}}\times \text{total }{\text{N}}_{\text{soybean}}$$

Percentage N in maize derived from transfer (% NDFT):

$$\%\text{NDFT = }{\text{N}}_{\text{transfer}}\times 100/\text{total }{\text{N}}_{\text{maize}}$$

The percent N derived from the atmosphere fixed by soybean (% Ndfa, Xiao et al., 2004):

%Ndfa = (1−atom% N15⁢excessmixed soybean/atom% N15⁢excessmono maize)⁢×100

where mono maize is the maize grown in monoculture. Mixed soybean is the soybean grown in soybean/maize intercropping.

### ^13^C Content Calculations

The content of ^13^C incorporated into different tissues (shoots, roots, or nodules) of soybean or maize was calculated based on the difference in atom% ^13^C excess of the labeled and non-labeled tissues as follows (Lu et al., 2002b):

C13=(atom %C13excesslabeled−atom % C13excessnon-labeled)×TC×100

where TC indicates the total tissue C content.

### Statistical Analyses

All of the data were analyzed to calculate mean and SE using Microsoft Excel 2007 (Microsoft Company, USA). SAS (SAS Institute Inc., Cary, NC, USA) was used for two-way (inoculation treatment and cropping system) or three-way (nutrient treatment, inoculation treatment, and cropping system) analysis of variance (ANOVA). Mean separation was conducted using the Duncan Multiple Range Test (DMRT) following two- or three-way ANOVA, and the least significant difference (LSD) for the two cropping systems or for two inoculation treatments.

## Results

### Plant Growth in the Greenhouse Experiment

The results showed that soybean and maize growth was significantly affected by N and P availability (*P* < 0.0001; **Table 1**, **Supplementary Table S1**). Biomasses of monocropped soybean and maize in the moderate N and P (MNMP) treatment increased by 68–173 and 311–502%, respectively, and those IS and maize biomasses increased by 53–158 and 247–766%, respectively, compared with their biomasses in the corresponding LN and P (LNLP) cultures (**Table 1**).

**Table 1:** Biomasses of soybean and maize inoculated with AMF and rhizobia under different N and P conditions in a greenhouse experiment.

	Biomass (g plant^-1^)			
		
Treatment		Monoculture		Intercropping	
			
		Soybean	Maize	Soybean	Maize
LNLP	-A-R	0.90 ± 0.02^g^	0.59 ± 0.06^e^	0.83 ± 0.06^e^	0.66 ± 0.08^e^
	+A-R	1.24 ± 0.08^ef^	1.08 ± 0.07^d^	1.26 ± 0.10^cd^	0.90 ± 0.04^d^
	+A+R	1.40 ± 0.03^de^	1.08 ± 0.07^d^	1.38 ± 0.13^c^	0.88 ± 0.06^d^
LNMP	-A-R	1.05 ± 0.05^fg^	0.81 ± 0.06^de^	0.87 ± 0.07^e^	0.81 ± 0.05^d^
	+A-R	1.60 ± 0.13^cd^	1.10 ± 0.08^d^	1.51 ± 0.13^c^	0.95 ± 0.07^d^
	+A+R	2.10 ± 0.09^b^	1.10 ± 0.08^d^	1.91 ± 0.07^b^	1.22 ± 0.10^d^
MNLP	-A-R	1.08 ± 0.08^fg^	0.74 ± 0.06^de^	0.97 ± 0.05^de^	1.02 ± 0.11^d^
	+A-R	1.48 ± 0.08^cde^	1.68 ± 0.13^c^	1.35 ± 0.11^c^	1.93 ± 0.09^c^
	+A+R	1.66 ± 0.09^c^	1.68 ± 0.13^c^	1.21 ± 0.15^cd∗^	2.01 ± 0.21^c^
MNMP	-A-R	1.51 ± 0.10^cd^	2.44 ± 0.07^b^	1.27 ± 0.08^cd^	2.29 ± 0.21^c^
	+A-R	3.39 ± 0.12^a^	6.48 ± 0.34^a^	3.24 ± 0.07^a^	5.77 ± 0.35^b^
	+A+R	3.65 ± 0.07^a^	6.48 ± 0.34^a^	3.35 ± 0.14^a^	7.60 ± 0.29^a∗^


*Data in the table are the mean of four replicates with standard error. Different letters indicate significant differences among nutrient treatments within a cropping system and crop species (Duncan’s multiple range test, *P* < 0.05). Asterisks indicate significant differences between monoculture and intercropped plants within a crop species and nutrient treatment (LSD test, *P* < 0.05). LNLP, low N and P; LNMP, low N and moderate P; MNLP, moderate N and low P; MNMP, moderate N and P.*

AM fungal and rhizobium inoculation produced great effects on soybean and maize growth. Inoculation with rhizobia and/or AMF dramatically increased biomass of soybean and maize (*P* < 0.0001; **Table 1**, **Supplementary Table S1**). Compared to uninoculated controls, in LNLP, soybean biomass in monoculture and intercropping systems increased by 38 and 52%, respectively, with only AMF inoculation, and by 55 and 66%, respectively, with co-inoculation, while maize biomass increases were 81 and 37%, respectively, with only AMF inoculation, and 81 and 33%, respectively, with co-inoculation. Moderate application of N and P amplified the effect of AM fungal and rhizobium inoculation, with monoculture and intercropped biomass increases compared to uninoculated controls of 124 and 156%, respectively, for soybean inoculated with AMF alone, 141 and 164%, respectively, for co-inoculated soybean, 165 and 152%, respectively, for maize only inoculated with AMF, and 165 and 233%, respectively, for co-inoculated maize.

Cropping system had a significant effect on soybean growth (*P* < 0.0001; **Table 1**; **Supplementary Table S1**). Biomasses of all soybean plants in intercropping treatments were lower than in monoculture across nutrient levels and inoculation treatments. Under moderate N and low P (MNLP) conditions, IS biomass was 27% less than in monoculture. Conversely, all maize plants in monoculture and intercropping systems responded positively to increased nutrient and inoculation treatments (**Table 1**; **Supplementary Table S1**). Yet, the only effect of intercropping observed for maize was when the biomass of IM was 17% higher than the biomass of monocultured maize in the MNMP and co-inoculation treatment.

**FIGURE 1:**
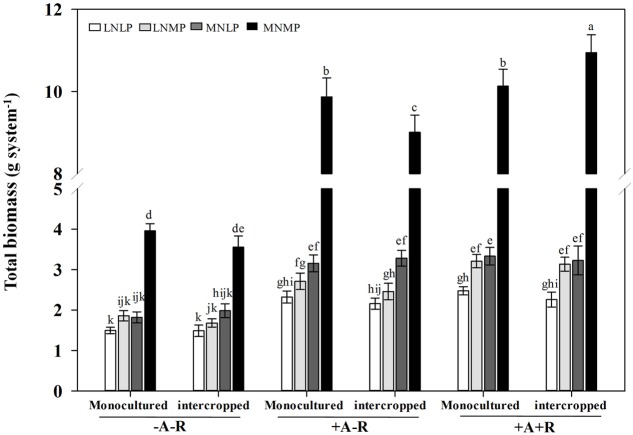
**Effects of inoculation with AMF and rhizobia on total biomasses of soybean and maize in a greenhouse experiment.** Data in the figure are the mean of four replicates with standard error. Different letters indicate significant differences among inoculation treatments (Duncan’s multiple range test, *P* < 0.05). -A-R: no inoculation; +A-R: sole inoculation with AMF; +A+R: co-inoculation with AMF and rhizobia. LNLP, low N and P; LNMP, low N and moderate P; MNLP, moderate N and low P; MNMP, moderate N and P.

In addition, total biomass of IM and soybean was also significantly affected by nutrient availability and inoculation treatments. Co-inoculation had positive effects on total biomass in both monoculture and intercropping systems, and moderate application of N and P amplified the effect of co-inoculation. Total biomass was obviously higher than those of sole maize and soybean in the MNMP and co-inoculation treatment (*P* < 0.0001; **Figure 1**; **Supplementary Table S1**).

### Plant N and P Contents in the Greenhouse Experiment

Nitrogen and P contents of all soybean and maize plants were significantly affected by N and P availability, along with AM fungal and rhizobium inoculation. This is consistent with the plant biomass results (**Table 1**). Moderate N and P application significantly enhanced N and P contents compared to low application rates regardless of inoculation treatment or cropping system (*P* < 0.0001; **Tables 2** and **3**; **Supplementary Table S1**). Both soybean and maize accumulated more N and P with MN and MP availability than those with LN and LP availability. Inoculations of soybean and maize with AMF and rhizobia significantly enhanced the contents of N and P in all cropping and nutrient treatments, except for the N and P contents of IM in the co-inoculation treatment reared in the LN treatment (*P* < 0.0001; **Tables 2** and **3**; **Supplementary Table S1**). With inoculation, the respective N contents of soybean and maize increased by 19–273 and 22–160%, and the respective P contents increased by 40–559 and 74–544% over non-inoculated controls. Moderate N and P application once again amplified the differences among inoculation treatments.

**Table 2:** N contents of soybean and maize inoculated with AMF and rhizobia under different N and P conditions in a greenhouse experiment.

	N content (mg plant^-1^)			
		
Treatment		Monoculture		Intercropping	
			
		Soybean	Maize	Soybean	Maize
LNLP	-A-R	12.57 ± 0.30^g^	5.32 ± 0.48^e^	10.53 ± 1.02^f^	6.94 ± 0.51^d^
	+A-R	18.27 ± 0.71^f^	8.14 ± 0.50^d^	19.32 ± 1.29^e^	8.49 ± 0.87^d^
	+A+R	20.63 ± 0.89^f^	8.14 ± 0.50^d^	19.53 ± 2.41^e^	8.01 ± 0.82^d^
LNMP	-A-R	11.66 ± 0.54^g^	6.30 ± 0.77^e^	9.69 ± 1.19^f^	6.70 ± 1.49^d^
	+A-R	26.14 ± 1.24^e^	10.12 ± 1.22^d^	25.51 ± 2.06^de^	8.19 ± 1.02^d^
	+A+R	42.43 ± 2.40^cd^	10.12 ± 1.22^d^	36.15 ± 2.11^bc^	10.99 ± 0.87^d^
MNLP	-A-R	28.27 ± 1.67^e^	11.68 ± 1.27^d^	25.07 ± 1.07^de^	15.09 ± 1.12^d^
	+A-R	41.83 ± 1.22^cd^	26.72 ± 2.06^c^	39.14 ± 2.53^b^	33.13 ± 2.65^c^
	+A+R	46.08 ± 2.04^c^	26.72 ± 2.06^c^	29.87 ± 5.36^cd∗^	31.51 ± 3.88^c^
MNMP	-A-R	37.71 ± 2.21^d^	36.36 ± 1.58^b^	32.04 ± 2.22^bcd^	33.53 ± 5.44^c^
	+A-R	77.98 ± 2.34^b^	73.18 ± 3.29^a^	72.78 ± 4.03^a^	73.10 ± 6.23^b^
	+A+R	87.34 ± 2.90^a^	73.18 ± 3.29^a^	72.80 ± 4.71^a∗^	87.32 ± 4.38^a∗^


*Data in the table are the mean of four replicates with standard error. Different letters indicate significant differences among nutrient treatments within a cropping system and crop species (Duncan’s multiple range test, *P* < 0.05). Asterisks indicate significant differences between monoculture and intercropped plants within a crop species and nutrient treatment (LSD test, *P* < 0.05). LNLP, low N and P; LNMP, low N and moderate P; MNLP, moderate N and low P; MNMP, moderate N and P.*

**Table 3:** P contents of soybean and maize inoculated with AMF and rhizobia under different N and P conditions in a greenhouse experiment.

	P content (mg plant^-1^)			
		
Treatment		Monoculture		Intercropping	
			
		Soybean	Maize	Soybean	Maize
LNLP	-A-R	0.53 ± 0.05^g^	0.27 ± 0.03^d^	0.54 ± 0.03^d^	0.36 ± 0.04^e^
	+A-R	0.95 ± 0.04^f^	0.82 ± 0.07^c^	1.01 ± 0.04^c^	0.66 ± 0.07^de^
	+A+R	1.03 ± 0.06^f^	0.82 ± 0.07^c^	1.11 ± 0.18^c^	0.72 ± 0.10^de^
LNMP	-A-R	1.40 ± 0.07^e^	0.86 ± 0.03^c^	1.08 ± 0.08^c∗^	1.02 ± 0.11^d^
	+A-R	2.78 ± 0.03^d^	1.94 ± 0.05^b^	2.88 ± 0.21^b^	1.76 ± 0.10^cd^
	+A+R	3.69 ± 0.16^c^	1.94 ± 0.05^b^	3.19 ± 0.17^b^	2.36 ± 0.08^c∗^
MNLP	-A-R	0.58 ± 0.11^g^	0.32 ± 0.07^d^	0.47 ± 0.07^d^	0.40 ± 0.06^e^
	+A-R	0.97 ± 0.07^f^	0.89 ± 0.11^c^	0.91 ± 0.04^c^	1.19 ± 0.16^d^
	+A+R	1.14 ± 0.10^ef^	0.89 ± 0.11^c^	0.67 ± 0.12^cd∗^	0.98 ± 0.13^d^
MNMP	-A-R	1.02 ± 0.10^f^	1.80 ± 0.28^b^	0.81 ± 0.11^c^	1.27 ± 0.29^d^
	+A-R	6.19 ± 0.22^b^	6.20 ± 0.27^a^	5.31 ± 0.55^a^	7.12 ± 0.65^b^
	+A+R	6.62 ± 0.22^a^	6.20 ± 0.27^a^	5.34 ± 0.36^a∗^	8.20 ± 1.09^a∗^


*Data in the table are the mean of four replicates with standard error. Different letters indicate significant differences among nutrient treatments within a cropping system and crop species (Duncan’s multiple range test, *P* < 0.05). Asterisks indicate significant differences between monoculture and intercropped plants within a crop species and nutrient treatment (LSD test, *P* < 0.05). LNLP, low N and P; LNMP, low N and moderate P; MNLP, moderate N and low P; MNMP, moderate N and P.*

The cropping system also significantly affected both N and P contents in soybean and maize (*P* < 0.0001 for soybean; *P* < 0.05 for maize; **Tables 2** and **3**; **Supplementary Table S1**). N and P contents of soybean plants had a decreasing trend, whereas those of maize plants showed an increasing trend from monoculture to intercropping. Furthermore, this difference in N and P contents between monocultured and IM/soybean tended to increase with increasing N and P availability, as well as, with AM fungal and rhizobium inoculation. Compared with the corresponding monocultured plants, the respective increases in the N and P contents of IM plants were 19 and 32%, and the decreases in the respective N and P contents of IS were 17 and 19% in the co-inoculation treatment with MNMP availability (**Tables 2** and **3**). In addition, relative to monocultured maize and soybean, with co-inoculation, there was a 22% increase in the P content of IM in LN and moderate P (LNMP), along with 35 and 42% decreases in N and P contents, respectively, of IS reared in MNLP.

### Mycorrhizal Colonization and Nodulation in the Greenhouse Experiment

In the greenhouse experiment, all uninoculated plants were non-mycorrhizal (results not shown). All inoculated plants were well colonized by AMF, with AM colonization ranging between 74 and 87% in soybean, and between 76 and 86% in maize (**Supplementary Figure S2**). Even so, an effect of nutrient levels on AM colonization was observed (*P* < 0.05; **Supplementary Table S1**). In soybean, AM colonization of monoculture plants in MNMP was 14.8% lower than in MNLP given only AM fungal inoculation.

**FIGURE 2:**
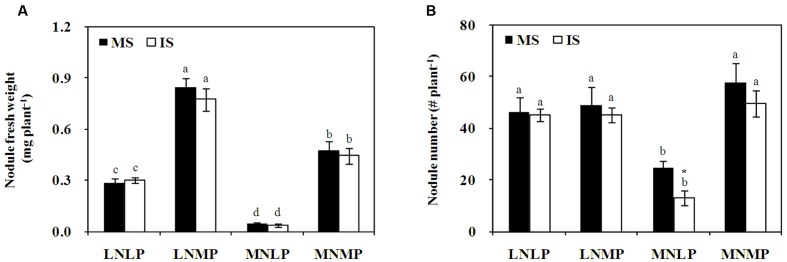
**Fresh weight** **(A)** and numbers **(B)** of nodules collected from soybean reared under different N and P conditions in a greenhouse experiment. Data in the figure are the mean of four replicates with standard error. Different letters indicate significant differences among nutrient levels (Duncan’s multiple range test, *P* < 0.05). Asterisks indicate significant differences between monoculture and intercropping within one nutrient level (LSD test, *P* < 0.05). **(A):** nodule fresh weight; **(B):** nodule number. MS, monoculture soybean; IS, intercropped soybean. LNLP, low N and P; LNMP, low N and moderate P; MNLP, moderate N and low P; MNMP, moderate N and P.

N and P application also affected soybean nodulation (*P* < 0.0001; **Figure 2A**; **Supplementary Table S2**). In comparison with the LNLP treatment, nodule fresh weights increased by 201 and 158% in LNMP, and by 70 and 48% in MNMP, whereas they decreased by 83 and 88% in MNLP for monocultured and IS, respectively. Nodule numbers also varied significantly among N and P treatments (*P* < 0.0001; **Figure 2B**; **Supplementary Table S2**), with decreases of 88 and 246% observed in MNLP relative to the number of nodules observed on soybean growing in LNLP under monocropping and intercropping conditions, respectively. Between the two cropping systems, there were no significant differences observed in nodule fresh weight or nodule number, except that the number of nodules on monocropped soybean was 88% higher than on IS growing with MNLP availability (**Figure 2B**).

### N_2_ Fixation and Nitrogen Transfer in the Labeling Experiment

To explore whether the growth differences observed between intercropped and the corresponding monocultured plants in co-inoculation treatments were due to N transfer through CMN after inoculation, the N transfer from soybean to maize was followed by ^15^N labeling in two compartment systems. As in the greenhouse experiment, inoculation with AMF and rhizobia significantly increased biomass and N content of soybean and maize irrespective of cropping system (*P* < 0.0001; **Supplementary Figure S3**; **Supplementary Table S3**). No significant difference in AM colonization was observed among treatments, except that colonization was lower in monocultured soybean inoculated only with AMF than it was in co-inoculated and IS and maize (Supplementary Figure S4; **Supplementary Table S3**). Biomass and N contents of maize plants were obviously higher when grown together with a neighboring soybean in co-inoculation treatments (*P* < 0.01; **Supplementary Figure S3**; **Supplementary Table S3**). In contrast, biomass and N content of IS showed the decreased trend compared with monocultured soybean (**Supplementary Figure S3**; **Supplementary Table S3**). As a consequence, soybean was, again, a weak competitor compared with maize in soybean/maize intercropping systems.

Biological nitrogen fixation was measured in the co-inoculation treatment. Soybean displayed a high N-fixing capacity, with more than 58% of its N being derived from the atmosphere (**Table 4**). This amounted to 13.4 mg plant^-1^ in the co-inoculation treatment. Petiole injection of ^15^N in the soybean donor was correlated with the appearance of the label in the maize receiver plants in the intercropping system. The ^15^N Petiole injection of soybean resulted in a higher N transfer from soybean to maize in both the AM fungal inoculation alone and the co-inoculation treatments. Although there was no significant difference in percentage of N transfer (% N_transfer_) between the AM fungal inoculation alone and co-inoculation treatments, the amounts of N transferred from soybean to maize were significantly different, with 1.3 and 2.0 mg plant^-1^ observed, respectively (**Table 4**). Co-inoculation with AMF and rhizobia enhanced N transfer from soybean to maize, and the percentage N in maize derived from transfer (% NDFT) increased from 11.1% with only AM fungal inoculation to 15.2% with co-inoculation (**Table 4**).

**Table 4:** N transfer and proportion of N fixed by soybean as affected by inoculation in a growth chamber labeling experiment

	+A-R	+A+R
% Ndfa		58.4 ± 6.8
Ndfa (mg plant^-1^)		13.4 ± 3.4
% N_transfer_	11.4 ± 1.0^a^	9.4 ± 1.9^a^
N transferred (mg plant^-1^)	1.3 ± 0.1^a^	2.0 ± 0.2^b^
% NDFT	11.1 ± 0.8^a^	15.2 ± 1.2^b^


*Data in the table are the mean of three replicates with standard error. Different letters indicate significant differences between inoculation treatments (LSD test, *P* < 0.05). % Ndfa: percent N derived from the atmosphere fixed by soybean; % N_transfer_: percentage N transferred from soybean to maize; N transferred: amount of N transferred from soybean to maize; % NDFT, percentage N in maize derived from transfer.*

### Carbon Allocation in the Labeling Experiment

**FIGURE 3:**
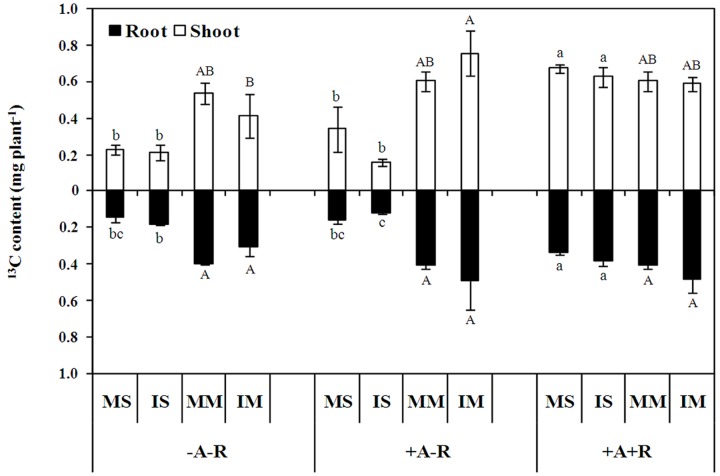
**^13^C recovery from shoots and roots of soybean and maize after 6 h labeling under a ^13^CO_2_-enriched atmosphere.** Data in the figure are the mean of three replicates with standard error. Different letters indicate significant differences among inoculation treatments within a crop species (Duncan’s multiple range test, *P* < 0.05). MS, monoculture soybean; IS, intercropped soybean. MM, monoculture maize; IM, intercropped maize. -A-R: no inoculation; +A-R: sole inoculation with AMF; +A+R: co-inoculation with AMF and rhizobia.

To evaluate the impacts of carbon allocation on the competitive abilities of maize and soybean in the intercropping system, pulse labeling of ^13^C was performed with soybean and maize plants in a walk-in growth chamber. The results showed that ^13^C content in monocultured and IS plants increased by 200 and 194% in shoots, and 130 and 113% in roots, respectively, with co-inoculation compared with uninoculated control plants (*P* < 0.0001; **Figure 3**; **Supplementary Table S3**). However, nodule ^13^C cost was similar in monocultured and IS, and accounted 7% of soybean root ^13^C content, respectively, in co-inoculation treatment. Interestingly, the ^13^C content of IS roots in the AM fungal inoculation treatment exhibited a 34% decrease compared with uninoculated controls, as well as, 75 and 69% decreases in shoots and roots, respectively, compared to the co-inoculation treatment (**Figure 3**). In contrast, the ^13^C content in IM shoots increased by 83% with AM fungal inoculation compared with uninoculated maize plants (**Figure 3**). These results indicate that photosynthate assimilation is stimulated by AM symbiosis in maize, and by rhizobial symbiosis in soybean, especially in the intercropping system.

## Discussion

In legume/cereal intercropping systems, legumes are generally weak competitors compared with cereals (Willey and Rao, 1980; Hauggaard-Nielsen and Jensen, 2001), which is often ascribed to differences in the root distributions of legumes and cereals, and the resulting differences in the ability of these crops to compete for soil N (Fan et al., 2006). In the maize/soybean intercropping system, the yield advantage of maize has been also reported (Tang et al., 2005; Meng et al., 2015). In the present study, we demonstrated that the high competitive ability of maize relative to soybean can be partly ascribed to the contribution of AMF and rhizobia. To our knowledge, this is the first report that the beneficial effects of intercropping on maize growth are due, at least in part, to allocation of carbon and nitrogen regulated by nodulation and mycorrhizal networks in the soybean/maize intercropping system.

Mycorrhizal colonization under high P conditions, and legume N fixation under high N conditions are often inhibited compared with these activities in P or N starved plants (Paynel et al., 2008; Breuillin et al., 2010; Balzergue et al., 2011; Wang et al., 2011). A previous study showed that co-inoculation with rhizobia and AMF significantly increases soybean growth in LP and/or LN conditions, but not with high P and high N nutrient availabilities (Wang et al., 2011). Therefore, in the present greenhouse experiments, medium N/P and LN/P treatments were employed to explore the effect of AMF and rhizobium inoculation on growth of IS and maize. The results showed that nodule fresh weight in MNMP was higher than in LNLP (**Figure 2A**). This indicates that an adequate nutrient supply is beneficial for nodule growth. In addition, both inoculation with AMF alone and co-inoculation with rhizobia promoted biomass, and increased the N and P contents of all soybean and maize plants compared with uninoculated controls regardless of nutrient status (**Tables 1**–**3**). Nevertheless, the largest effect of inoculation was found in MNMP relative to the other nutrient treatments, suggesting that the inoculation effect is dependent upon nutrient status. Another interesting phenomenon observed herein was that IM produced a yield advantage only in MNMP when co-inoculated with AMF and rhizobia (**Tables 1**–**3**). This implies that the contributions of AMF and rhizobia to crop advantages in intercropping are also closely related to nutrient status, which might in the end be ascribed to the regulation of N transfer and carbon allocation between maize and soybean.

It has been well reported that N can be transferred from legumes to cereals in intercropping systems by indirect or direct pathways (He et al., 2004, 2005, 2009; Xiao et al., 2004). Indirect pathways transfer N released from dead and decayed legume tissues, and from legume root exudates to the rhizosphere, where they are taken up from the soil solution by cereal roots or hyphae. Direct N transfer is mediated by CMN between coexisting legumes and cereals (He et al., 2004; Paynel et al., 2008). In the present work, petiole injection of ^15^N into soybean was used to detect direct N transfer from soybean to neighboring maize plants along an N concentration gradient via CMNs. Dividing cultures with a double nylon mesh and an air gap were designed to prevent the diffusion of nutrients, and allow passage of hyphae but not roots. The results showed that AMF inoculation alone resulted in a net 11.4% ^15^N transfer, and co-inoculation of AMF and rhizobia led to a 54% increase in amount of ^15^N transferred from soybean to maize (**Table 1**). This considerable difference in the amount of N transferred between single and dual inoculation treatments indicates that mycorrhizae and rhizobia act together to enhance N fluxes from mycorrhizal soybean with nodules to mycorrhizal maize, and that increased N transfer partly results from symbiotic N fixation (**Table 4**). Therefore, increases in the biomass of maize are due to increases in N content resulting from intercropping with soybean and in the presence of both AMF and rhizobia.

The competitive advantage of maize when intercropped with soybean might also be due to changes in carbon allocation in these plants as affected by AM and rhizobium colonization. Rhizobial and AM symbioses typically consume 4–16% of plant photosynthetic carbon in order to maintain symbiont growth, activity and reserves, with co-colonization possibly leading to additive effects on C costs (Johnson et al., 2002; Kaschuk et al., 2009). Simultaneously, carbon sinks of rhizobial and AM symbioses can stimulate photosynthesis (Kaschuk et al., 2009). It is estimated that the rate of photosynthesis can increase by 28 and 14% in response to rhizobial and AM fungal inoculation, respectively, and by 51% with co-inoculation (Kaschuk et al., 2009). In the intercropping system, carbon can flow through CMNs (Walder et al., 2012). In the present study, IM with AMF alone contained more shoot ^13^C than uninoculated maize, while soybean displayed the opposite effect (**Figure 3**). This indicates that AM fungal inoculation stimulates the capacity of maize to assimilate photosynthate, while IS invests more carbon into CMNs. On the other hand, with co-inoculation, the ^13^C content was highest for soybean in both shoots and roots compared with uninoculated soybeans or those inoculated with AMF alone, irrespective of cropping system, while no significant changes in maize ^13^C content were found (**Figure 3**). Taken together, these results suggest that carbon fixation is stimulated by rhizobial symbiosis in soybean, and soybean plants, which are typically not C-limited, were then able to match increases in nutrient acquisition from roots with higher N content under co-inoculation conditions compared to instances of no symbiont inoculation or inoculation with AMF alone (**Supplementary Figure S3**). Furthermore, no differences in soybean biomass between uninoculated plants and those with AMF inoculation alone could be ascribed to higher C costs associated with AM fungal inoculation. Therefore, the loss of biomass in IS relative to monocultures might be attributable to increased N transfer to maize via CMNs in co-inoculation treatments. This still needs to be further clarified by quantitative analysis of carbon costs of extracted AMF hyphae in future studies.

Previous studies have shown that the C_3_ plant flax acquires more N and P provided by the CMN with little carbon costs, whereas the C_4_ plant sorghum invests more carbon with little N and P return (Walder et al., 2012). Therefore, the net benefit for flax is much greater than for sorghum in flax/sorghum mixed cultures. This is inconsistence with the present results, in which the C_4_ plant maize shows a higher competitive advantage than the C_3_ plant soybean (**Table 1**; **Supplementary Figure S3**). This can be explained by differences among these crop species. Soybean as legume plant can form tripartite symbiotic associations with rhizobia and AMF simultaneously (Wang et al., 2011). In the present study, rhizobial symbiosis stimulates the assimilation capacity of photosynthetic carbon in the C_3_ plant, soybean, which then invests more carbon into CMNs. Conversely, the C_4_ plant maize invests little carbon, yet receives 15% of its N from soybean through N transfer via CMNs.

Soybean is a weak competitor compared with maize in soybean/maize intercropping system. However, the overall productivity of the soybean/maize intercropping system is significantly higher than those of sole maize and soybean when co-inoculated with AMF and rhizobia under moderate nutrient supply conditions (**Figure 1**). Soybean and maize can be naturally inoculated by indigenous AM fungi and rhizobia in the field. Generally, indigenous rhizobia have low nodulation activities than those of inoculation (Qin et al., 2012). Therefore, effective rhizobium inoculation combined with indigenous AM fungi not only increases N uptake through symbiotic N2 fixation but also enhances N transfer by CMNs, which could subsequently contribute to improve intercropping advantages in soybean/maize intercropping system in the field.

## Conclusion

These results showed that maize has a competitive advantage over soybean in soybean/maize intercropping system. The important aspect of this work is the demonstration that the growth advantage of maize is due to increased N acquisition from soybean via CMNs and a relatively low carbon investment into CMNs by maize growing with soybean under conditions favoring symbioses with both rhizobia and AMF.

## Author Contributions

XW and HL conceived the experiments. XW, JS, GW, LS, and DZ designed and performed the experiments. XW, JS and HL drafted the manuscript. XW and HL edited the manuscript. All authors approved the final version.

## Conflict of Interest Statement

The authors declare that the research was conducted in the absence of any commercial or financial relationships that could be construed as a potential conflict of interest.

## Abbreviations

AMF: Arbuscular mycorrhizal fungi
CMN: common mycorrhizal network
IM: intercropped maize
IS: intercropped soybean
LN: low N
LP: low P
MM: monoculture maize
MN: medium N
MP: medium P
MS: monoculture soybean
N: nitrogen
P: phosphorus
