# Supplementary material for: Allocation of Nitrogen and Carbon Is Regulated by Nodulation and Mycorrhizal Networks in Soybean/Maize Intercropping System
Source: Front Plant Sci. 2016 Dec 16;7:1901. doi: 10.3389/fpls.2016.01901 (PMC5160927; doi:10.3389/fpls.2016.01901)
Supplement: Supplementary file 1 [file Table_1.DOC]

Supplementary Material:

**Allocation of nitrogen and carbon is regulated by nodulation and mycorrhizal networks in soybean/maize intercropping system**

Guihua Wang^1^, Lichao Sheng^2^, Dan Zhao^2^, Jiandong Sheng^2^*, Xiurong Wang^1^* and Hong Liao^3^

1. State Key Laboratory for Conservation and Utilization of Subtropical Agro-bioresources, Root Biology Center, South China Agricultural University, Guangzhou, China; 2. Xinjiang Key Laboratory of Soil and Plant Ecological Processes, College of Grassland and Environmental Sciences, Xinjiang Agricultural University, Urumqi, China; 3. Root Biology Center, Fujian Agriculture and Forestry University, Fuzhou, China

***Correspondences:**

**Xiurong Wang**

[xrwang@scau.edu.cn](mailto:hliao@scau.edu.cn);

**Jiandong Sheng**

[sjd_2004@126.com](mailto:sjd_2004@126.com)

**Table S1.** F values of a three-way ANOVA testing for effects of nutrient levels (N), inoculation treatments (I), cropping systems (C), or their interactions on biomass, N and P contents, and AM colonization of soybean and maize in a greenhouse experiment. Statistically significant effects are indicated by asterisks: *, *p* ≤ 0.05; **, *p* ≤ 0.01; ***, *p* ≤ 0.001; ns: not significant.

| Species | | | F values for | | | N | | I | | | C | | N×I | | N×C | | | | I×C | N×I ×C |
| --- | --- | --- | --- | --- | --- | --- | --- | --- | --- | --- | --- | --- | --- | --- | --- | --- | --- | --- | --- | --- |
| Soybean | | | Biomass | | | 387.29*** | | 289.98*** | | | 22.12*** | | 49.37*** | | 2.06 ns | | | | 0.85 ns | 0.50 |
|  | | | N content | | | 440.61*** | | 222.13*** | | | 25.42*** | | 32.74*** | | 3.59* | | | | 5.97** | 0.49 ns |
|  | | | P content | | | 506.73*** | | 322.78*** | | | 17.16*** | | 97.98*** | | 5.98** | | | | 2.99* | 1.36 ns |
|  | | | AM colonization | | | 4.37** | | 3.39 ns | | | 0.87 ns | | 0.46 ns | | 0.32 ns | | | | 0.15 ns | 0.16 ns |
| Maize | | | Biomass | | | 1222.17*** | | 279.75*** | | | 0.48 ns | | 118.08*** | | 2.09 ns | | | | 8.95 ** | 6.44*** |
|  | | | N content | | | 657.79*** | | 111.20*** | | | 5.02* | | 38.33 *** | | 1.47 ns | | | | 1.78 ns | 1.83 ns |
|  | | | P content | | | 465.29*** | | 165.24*** | | | 6.91* | | 65.12*** | | 3.58* | | | | 3.72* | 3.78** |
|  | | | AM colonization | | | 3.94* | | 0.79 ns | | | 0.90 ns | | 0.50 ns | | 0.13 ns | | | | 0.79 ns | 0.50 ns |
| Soybean +Maize | | | Total biomass | | | 1609.21*** | | 550.26*** | | | 2.10 ns | | 161.55*** | | 0.63 ns | | | | 2.92 ns | 3.82** |
|  |  | | | | | | | | | | | | | | | | |  | | |
|  |  | | | | | | | | | | | | | | | | |  | | |
|  |  |  | |  |  | |  | |  |  | |  | |  | |  |  | | | |
|  |  | | | | | | | | | | | | | | | |  | | | |
|  |  | | | | | | | | | | | | | | | |  | | | |
|  |  | | | | | | | | | | | | | | | |  | | | |
|  |  | | | | | | | | | | | | | | | |  | | | |
|  |  | | | | | | | | | | | | | | | |  | | | |
|  |  | | | | | | | | | | | | | | | |  | | | |
|  |  | | | | | | | | | | | | | | | |  | | | |
