# Supplementary material for: Allocation of Nitrogen and Carbon Is Regulated by Nodulation and Mycorrhizal Networks in Soybean/Maize Intercropping System
Source: Front Plant Sci. 2016 Dec 16;7:1901. doi: 10.3389/fpls.2016.01901 (PMC5160927; doi:10.3389/fpls.2016.01901)
Supplement: Supplementary file 2 [file Table_2.DOC]

**Table S2.** F values of a two-way ANOVA testing for effects of nutrient levels (N), cropping systems (C) or their interactions on nodulation parameters of soybean in a greenhouse experiment. Statistically significant effects are indicated by asterisks: *, *p* ≤ 0.05; **, *p* ≤ 0.01; ***, *p* ≤ 0.001; ns: not significant.

| Species | F values for | N | C | N×C |
| --- | --- | --- | --- | --- |
| Soybean | Nodule fresh weight | 133.66*** | 0.77 ns | 0.47 ns |
|  | Nodule number | 19.87*** | 3.11 ns | 0.46 ns |
