# Supplementary material for: Allocation of Nitrogen and Carbon Is Regulated by Nodulation and Mycorrhizal Networks in Soybean/Maize Intercropping System
Source: Front Plant Sci. 2016 Dec 16;7:1901. doi: 10.3389/fpls.2016.01901 (PMC5160927; doi:10.3389/fpls.2016.01901)
Supplement: Supplementary file 3 [file Table_3.DOC]

**Table S3.** F values of a two-way ANOVA testing for effects of inoculation treatments (I), cropping systems (C) or their interactions on biomass, N content, AM colonization, and ^13^C content of soybean and maize in a growth chamber labeling experiment. Statistically significant effects are indicated by asterisks: *, *p* ≤ 0.05; **, *p* ≤ 0.01; ***, *p* ≤ 0.001; ns: not significant.

| Species | F values for | I | C | I×C |
| --- | --- | --- | --- | --- |
| Soybean | Biomass | 19.66*** | 2.20 ns | 0.47 ns |
|  | N content | 40.91*** | 4.28 ns | 0.55 ns |
|  | AM colonization | 3.87 ns | 2.86 ns | 0.03 ns |
|  | Shoot ^13^C content | 32.90*** | 2.88 ns | 1.18 ns |
|  | Root ^13^C content | 75.31*** | 0.68 ns | 3.80 ns |
| Maize | Biomass | 8.32** | 9.67** | 0.44 ns |
|  | N content | 9.56** | 14.54** | 1.73 ns |
|  | AM colonization | 1.43 ns | 3.15 ns | 1.43 ns |
|  | Shoot ^13^C content | 3.08 ns | 0.01 ns | 1.44 ns |
|  | Root ^13^C content | 0.95 ns | 0.13 ns | 0.78 ns |
