# Supplementary material for: Allocation of Nitrogen and Carbon Is Regulated by Nodulation and Mycorrhizal Networks in Soybean/Maize Intercropping System
Source: Front Plant Sci. 2016 Dec 16;7:1901. doi: 10.3389/fpls.2016.01901 (PMC5160927; doi:10.3389/fpls.2016.01901)
Supplement: Supplementary file 4 [file Presentation_1.PPT]

## Slide 1
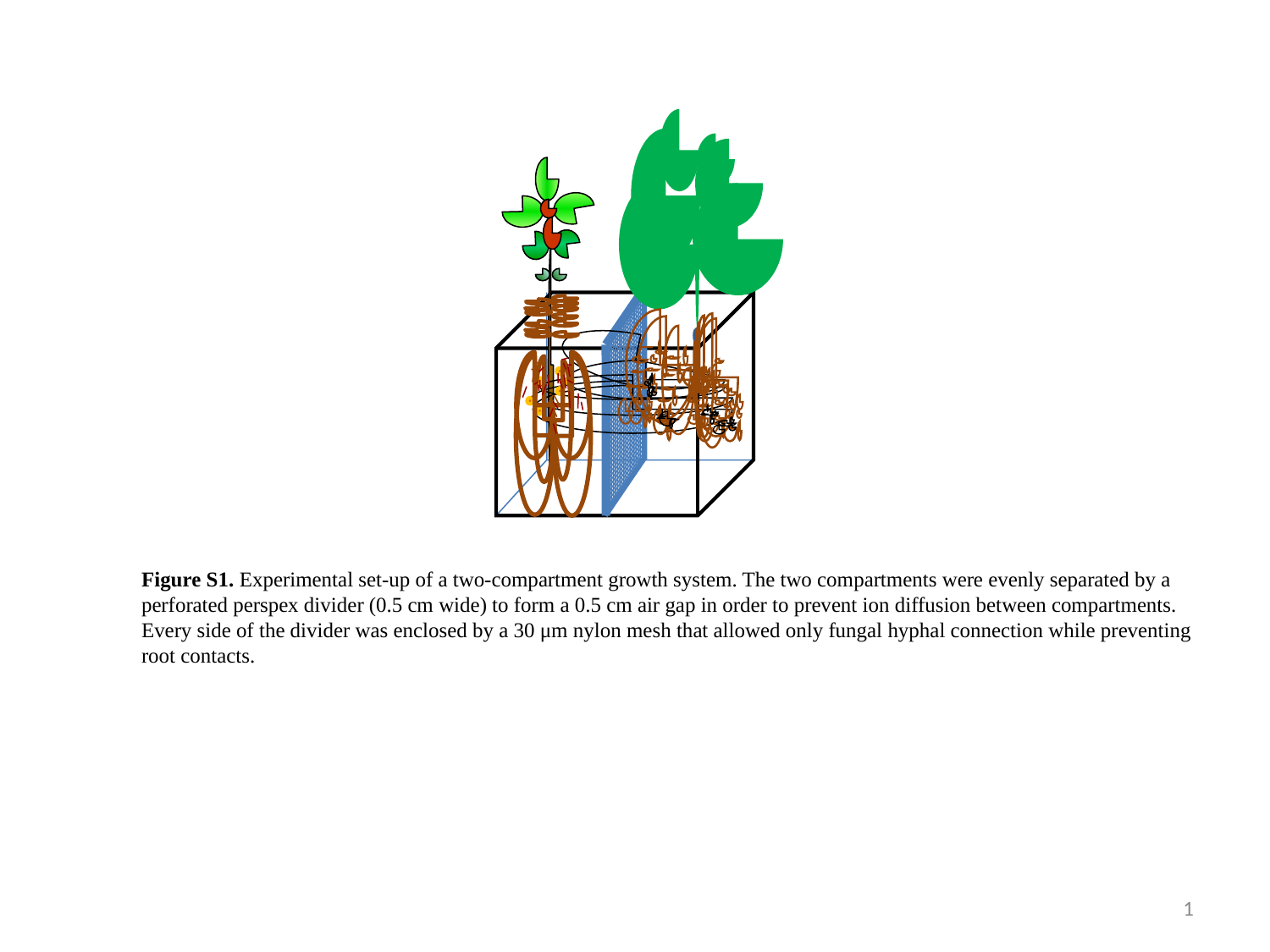

Figure S1. Experimental set-up of a two-compartment growth system. The two compartments were evenly separated by a perforated perspex divider (0.5 cm wide) to form a 0.5 cm air gap in order to prevent ion diffusion between compartments. Every side of the divider was enclosed by a 30 μm nylon mesh that allowed only fungal hyphal connection while preventing root contacts.
<number>
