# Supplementary material for: Allocation of Nitrogen and Carbon Is Regulated by Nodulation and Mycorrhizal Networks in Soybean/Maize Intercropping System
Source: Front Plant Sci. 2016 Dec 16;7:1901. doi: 10.3389/fpls.2016.01901 (PMC5160927; doi:10.3389/fpls.2016.01901)
Supplement: Supplementary file 5 [file Presentation_2.pptx]

## Slide 1
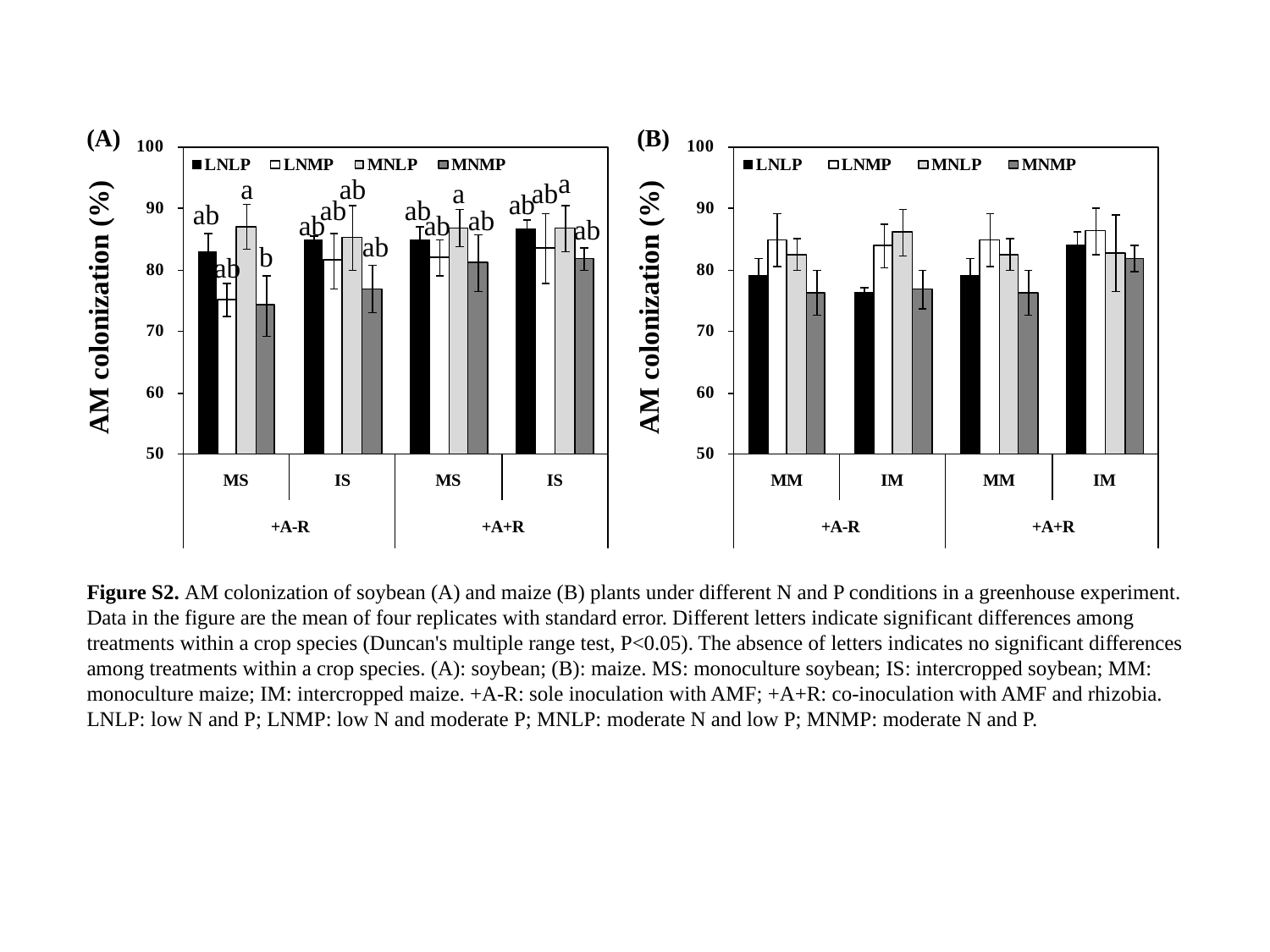

(A)
(B)
 a
 a
 ab
 a
 ab
 ab
 ab
 ab
 ab
 ab
 ab
 ab
 ab
 ab
 b
 ab
AM colonization (%)
AM colonization (%)
Figure S2. AM colonization of soybean (A) and maize (B) plants under different N and P conditions in a greenhouse experiment.
Data in the figure are the mean of four replicates with standard error. Different letters indicate significant differences among treatments within a crop species (Duncan's multiple range test, P<0.05). The absence of letters indicates no significant differences among treatments within a crop species. (A): soybean; (B): maize. MS: monoculture soybean; IS: intercropped soybean; MM: monoculture maize; IM: intercropped maize. +A-R: sole inoculation with AMF; +A+R: co-inoculation with AMF and rhizobia. LNLP: low N and P; LNMP: low N and moderate P; MNLP: moderate N and low P; MNMP: moderate N and P.
