# Supplementary material for: Allocation of Nitrogen and Carbon Is Regulated by Nodulation and Mycorrhizal Networks in Soybean/Maize Intercropping System
Source: Front Plant Sci. 2016 Dec 16;7:1901. doi: 10.3389/fpls.2016.01901 (PMC5160927; doi:10.3389/fpls.2016.01901)
Supplement: Supplementary file 6 [file Presentation_3.pptx]

## Slide 1
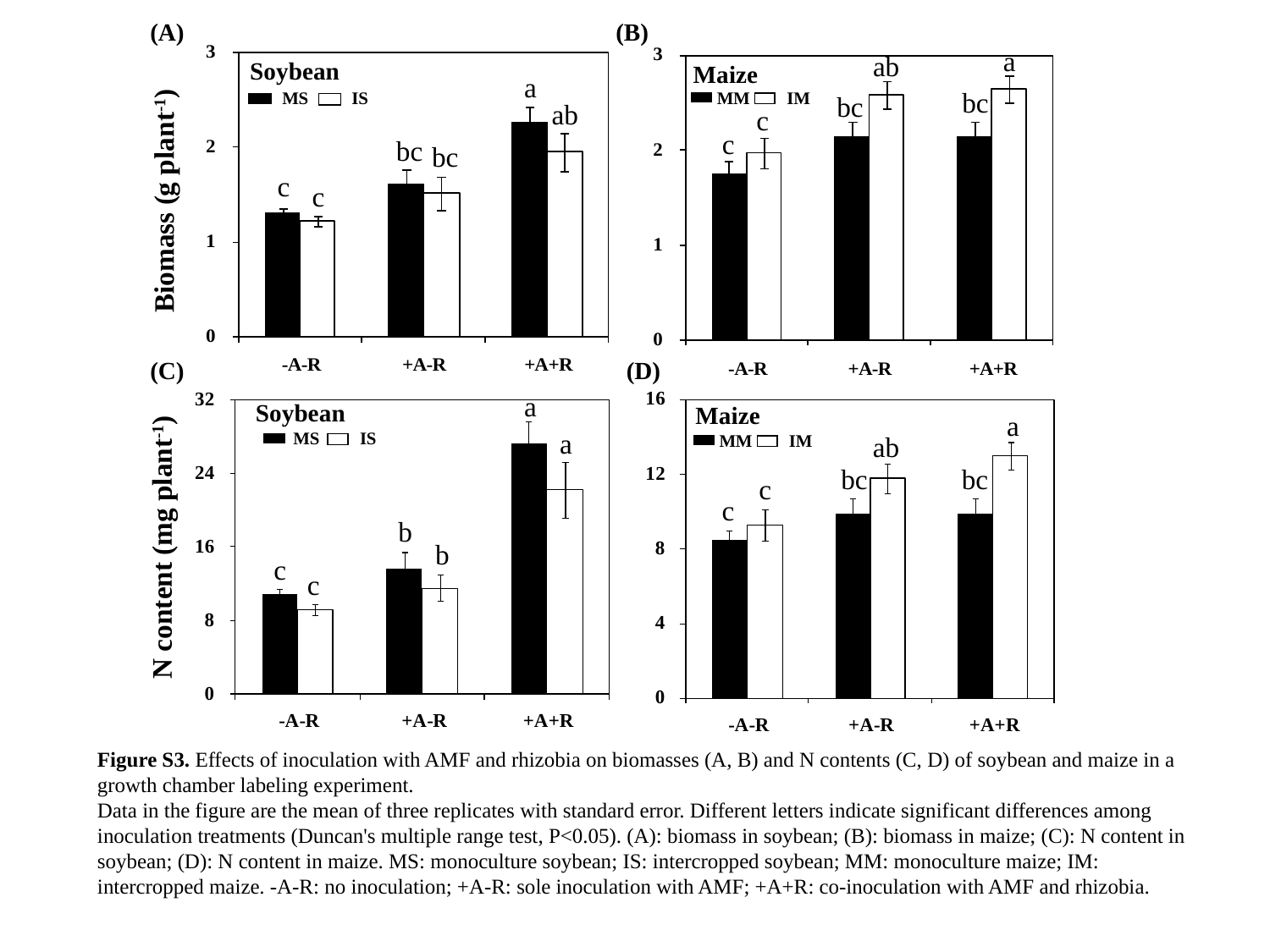

(A)
(B)
 a
 ab
Maize
MM
 IM
 bc
bc
 c
c
Soybean
MS
 IS
 a
 ab
 bc
 bc
 c
 c
 Biomass (g plant-1)
 a
Soybean
Maize
 a
a
MS
 IS
MM
 IM
 ab
 bc
 bc
 c
 c
 b
 b
 N content (mg plant-1)
 c
 c
(C)
(D)
Figure S3. Effects of inoculation with AMF and rhizobia on biomasses (A, B) and N contents (C, D) of soybean and maize in a growth chamber labeling experiment.
Data in the figure are the mean of three replicates with standard error. Different letters indicate significant differences among inoculation treatments (Duncan's multiple range test, P<0.05). (A): biomass in soybean; (B): biomass in maize; (C): N content in soybean; (D): N content in maize. MS: monoculture soybean; IS: intercropped soybean; MM: monoculture maize; IM: intercropped maize. -A-R: no inoculation; +A-R: sole inoculation with AMF; +A+R: co-inoculation with AMF and rhizobia.
