# Supplementary material for: Allocation of Nitrogen and Carbon Is Regulated by Nodulation and Mycorrhizal Networks in Soybean/Maize Intercropping System
Source: Front Plant Sci. 2016 Dec 16;7:1901. doi: 10.3389/fpls.2016.01901 (PMC5160927; doi:10.3389/fpls.2016.01901)
Supplement: Supplementary file 7 [file Presentation_4.pptx]

## Slide 1
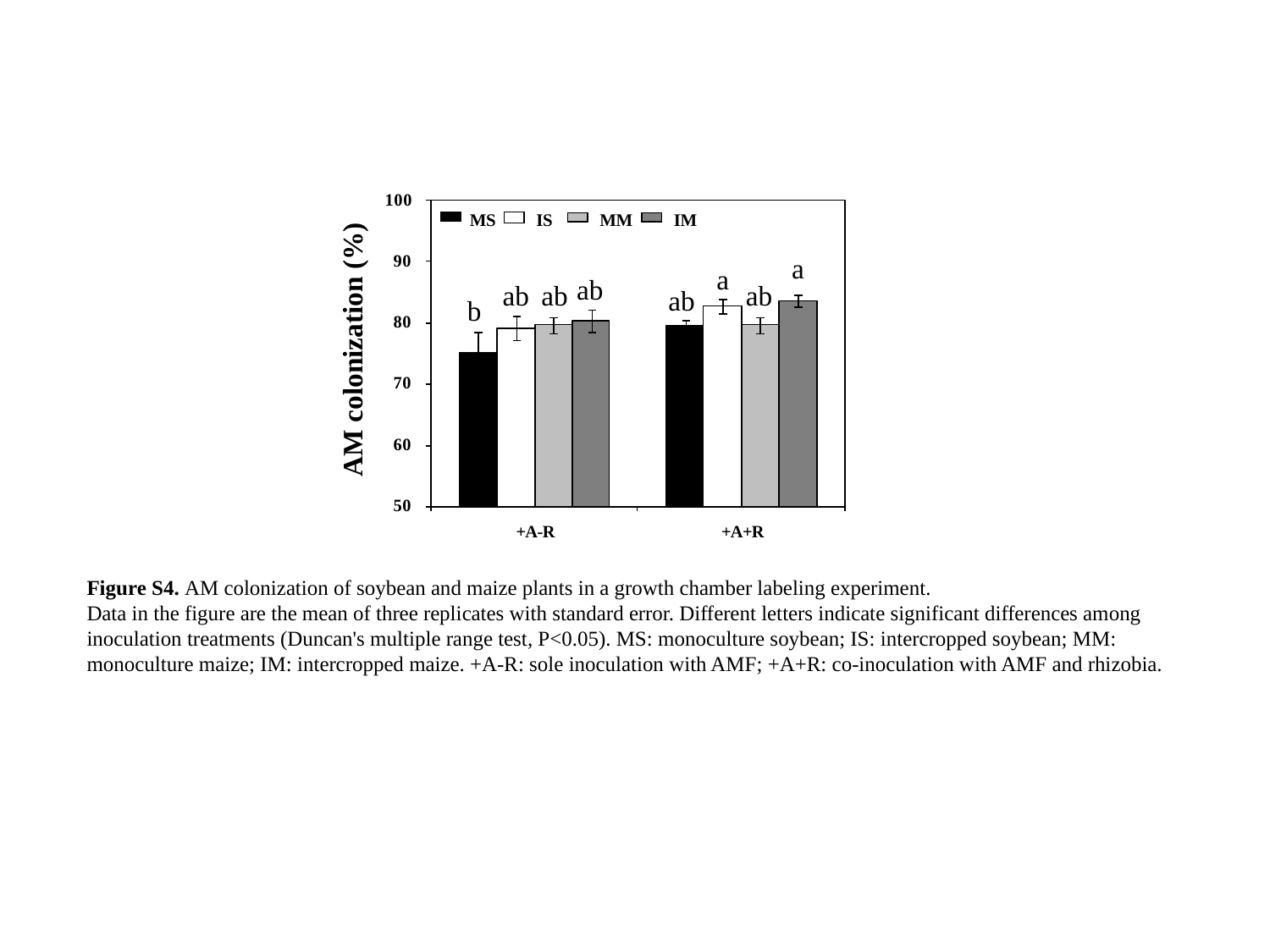

MS
 IS
 MM
 IM
 a
 a
 ab
 ab
 ab
 ab
 ab
b
AM colonization (%)
Figure S4. AM colonization of soybean and maize plants in a growth chamber labeling experiment.
Data in the figure are the mean of three replicates with standard error. Different letters indicate significant differences among inoculation treatments (Duncan's multiple range test, P<0.05). MS: monoculture soybean; IS: intercropped soybean; MM: monoculture maize; IM: intercropped maize. +A-R: sole inoculation with AMF; +A+R: co-inoculation with AMF and rhizobia.
